# Use of Feeding Tubes Among Hospitalized Older Adults With Dementia

**DOI:** 10.1001/jamanetworkopen.2024.60780

**Published:** 2025-02-20

**Authors:** Anne-Marie Hartford, Wenshan Li, Danial Qureshi, Robert Talarico, Stephen G. Fung, Shirley H. Bush, Genevieve Casey, Sarina R. Isenberg, Colleen Webber, Peter Tanuseputro

**Affiliations:** 1 School of Rehabilitation Therapy, Queen’s University, Kingston, Ontario, Canada; 2 Department of Clinical Epidemiology, Ottawa Hospital Research Institute, Ottawa, Ontario, Canada; 3 Nuffield Department of Population Health, University of Oxford, Oxford, United Kingdom; 4 ICES uOttawa, Ottawa Hospital Research Institute, Ottawa, Ontario, Canada; 5 Bruyère Research Institute, Ottawa, Ontario, Canada; 6 Division of Palliative Care, Department of Medicine, University of Ottawa, Ottawa, Ontario, Canada; 7 Division of Geriatrics, Department of Medicine, University of Ottawa, Ottawa, Ontario, Canada; 8 Department of Family Medicine and Primary Care, University of Hong Kong, Hong Kong

## Abstract

**Importance:**

Although dementia is a contraindication for feeding tube placement, guidelines recommending against its use are inconsistently followed, and factors associated with its use are unclear.

**Objective:**

To describe the incidence of feeding tube placement among hospitalized older adults (aged ≥65 years) with dementia and their health outcomes during and after hospitalization and to identify factors associated with placement of feeding tubes (ie, gastrostomy, gastrostomy-jejunostomy, and jejunostomy tubes).

**Design, Setting, and Participants:**

This population-based retrospective cohort study was conducted using a linked database in Ontario, Canada. Older adults who were diagnosed with dementia prior to a hospitalization between April 1, 2014, and March 31, 2018, were included. Data analyses were completed between October 2021 and November 2024.

**Exposures:**

Sociodemographic characteristics, health profiles, functional status, and advanced directives.

**Main Outcomes and Measures:**

Whether individuals received a feeding tube insertion (ie, gastrostomy, gastrostomy-jejunostomy, or jejunostomy tube), as identified by Ontario Health Insurance Plan billing codes.

**Results:**

Among 143 331 older adults with dementia (83 536 [58.3%] female; mean [SD] age, 83.8 [7.5] years), 1312 (0.9%) received a feeding tube in hospital and 142 019 (99.1%) did not. During hospitalization, feeding tube recipients stayed longer in hospital (mean [SD] stay, 65.6 [120.8] vs 14.8 [35.2] days for nonrecipients) and were more likely to be admitted to the intensive care unit (557 [42.5%] vs 14 423 [10.2%] of nonrecipients) or to die in hospital (294 [22.4%] vs 14 698 [10.3%] of nonrecipients). Within 1 year of discharge, 509 of 1018 feeding tube recipients (50.0%) died compared with 36 162 of 127 321 nonrecipients (28.4%). Among recipients of home care and long-term care residents, regression modeling showed that having swallowing problems (odds ratio [OR], 2.22; 95% CI, 1.99-2.49) and greater functional impairments (OR, 2.75; 95% CI, 1.80-4.20) were associated with increased odds of receiving a feeding tube, while being female (OR, 0.66; 95% CI, 0.52-0.84), older (OR for every 5-year increase in age, 0.75; 95% CI, 0.70-0.81), having a do-not-resuscitate directive (OR, 0.38; 95% CI, 0.31-0.47), and living in rural settings (OR, 0.38; 95% CI, 0.22-0.66) were associated with reduced odds.

**Conclusions and Relevance:**

In this cohort study of hospitalized individuals with dementia, feeding tube insertion was not associated with improved survival or postdischarge outcomes. Factors that were (or were not) associated with feeding tube placement were sometimes misaligned with best practice guidelines. Goals of care conversations, alternative intervention options, and improved clinical protocols are recommended.

## Key Points

**Question:**

What is the incidence of feeding tube placement in hospitalized adults with dementia and what health outcomes and factors are associated with feeding tube use?

**Findings:**

In this cohort study of 143 331 older adults (aged ≥65 years) with dementia, 1312 (0.9%) received a feeding tube while hospitalized, with recipients having higher use of health care resources and increased likelihood of in-hospital death after discharge. Being male or younger and having swallowing problems and greater functional impairments were associated with increased odds of feeding tube use, while having a do-not-resuscitate directive and living in rural settings were associated with reduced odds.

**Meaning:**

These findings suggest that health care professionals should carefully consider the appropriateness of feeding tube use among individuals with dementia and factors that influence their decisions.

## Teaser

This cohort study describes the incidence of feeding tube placement among hospitalized older adults with dementia, their health outcomes during and after hospitalization, and factors associated with feeding tube placement.

## Introduction

Dementia refers to a group of disorders related to cognitive decline and neurodegeneration, ultimately affecting everyday functioning.^1,2^ Older individuals with more advanced stages of dementia often develop issues with chewing and swallowing.^3,4,5^ A previous study^6^ reported 86% of nursing home residents experienced eating problems, while a scoping review^7^ reported prevalence of dysphagia as high as 93%. Consequently, family caregivers and health care professionals may need to decide whether to use a percutaneous endoscopic gastrostomy (PEG) feeding tube, which is inserted into the stomach through an incision in the abdomen. However, use of PEG tubes poses risks of developing pressure ulcers and aspiration pneumonia^8,9,10^ and has been considered of low value among individuals with dementia, with previous work showing these interventions were not associated with improved quality of life or lengthened survival time.^9,11,12,13,14,15,16,17,18,19,20^

Previous studies primarily focused on nursing home residents in the US with advanced dementia and reported that feeding tube use was associated with no improvements in health, with worse survival, and with greater use of health care resources.^11,12,15,21,22,23^ Notably, current guidelines from the Canadian Geriatrics Society,^24^ American Geriatrics Society,^25^ European Society for Clinical Nutrition and Metabolism,^26^ and the Canadian Choosing Wisely Campaign^27^ clearly recommend that PEG tubes should not be offered to individuals with advanced dementia. However, many previous studies were limited to individuals with advanced dementia, and no previous population-based study, to our knowledge, has examined feeding tube use and outcomes among community-dwelling individuals with dementia. Anecdotally, feeding tube placement still occurs for individuals with dementia, despite existing recommendations, yet gaps exist in the population-level incidence of feeding tube use and factors associated with its use.

Given these knowledge gaps, we sought to describe the incidence of feeding tube placement among hospitalized older adults (aged ≥65 years) with dementia in Ontario, Canada, regardless of dementia severity or place of residence. We also aimed to verify whether individuals with dementia who received a feeding tube (henceforth referred to as *feeding tube recipients*) had better or worse in-hospital and postdischarge outcomes than those who did not (henceforth referred to as *feeding tube nonrecipients*). Finally, we sought to identify factors associated with receipt of feeding tubes among the subgroups of hospitalized older adults with dementia who were receiving home care or admitted to long-term care (LTC) to identify reasons for feeding tube placement despite lack of demonstrated benefit.

## Methods

### Study Design, Setting, and Data Sources

We conducted a population-based retrospective cohort study in Ontario using linked databases held at ICES, an independent nonprofit research institute whose legal status under section 45 of Ontario’s Personal Health Information Protection Act allows it to collect and analyze health care and demographic data, without consent, for health system evaluation and improvement. Given this, this study did not require further ethics approval. Our study population included all adults with dementia 65 years or older at the time of admission to an acute care hospital and discharged between April 1, 2014, and March 31, 2018. Individuals’ first hospitalization within the study period served as their index hospitalization, with date of admission as the index date. Individuals were excluded if they were ineligible for the Ontario Health Insurance Plan (OHIP) at the index date or had missing or invalid birth date, sex, or death date. Detailed information about each dataset is provided in eTable 1 in Supplement 1. This study followed the Strengthening the Reporting of Observational Studies in Epidemiology (STROBE) reporting guideline.

Physician-diagnosed dementia was identified using a previously validated algorithm and data on physician claims from the OHIP; prescription claims from the Ontario Drug Benefit; hospitalizations from the Canadian Institute of Health Information’s Discharge and Abstract database; same-day surgical procedures from the Canadian Institute of Health Same Day Surgery database; and age from the Registered Persons Database. eMethods in Supplement 1 presents detailed definitions.^28^ We identified 2 subcohorts who were receiving publicly funded home care or LTC based on receipt of a Resident Assessment Instrument in Home Care (RAI-HC) or Resident Assessment Instrument in Long-Term Care (RAI-MDS) within the 6 months prior to the index hospitalization. In Ontario, the RAI-HC is a standardized routine assessment provided to home-care recipients every 6 months, and the RAI-MDS is a standardized routine assessment provided to LTC residents every 3 months. They assess individuals’ functional status, health profiles, service use, advance directives, and caregiver information (eTable 2 in Supplement 1). The assessment closest to the admission date of their index hospitalization was selected. The interrater reliabilities of items on both assessments are high, and the disease diagnoses items have been validated.^29,30,31^

### Receipt of Feeding Tubes

Receipt of a feeding tube was defined using a combination of Canadian Classification of Health Interventions codes and OHIP fee codes billed by physicians indicating insertion of a gastrostomy, gastrostomy-jejunostomy, or jejunostomy tube during index hospitalization. Further details are given in eTable 3 in Supplement 1.^32^

### Cohort Characteristics

We described the following characteristics of all individuals in this cohort: age at admission, sex, neighborhood income quintile (based on postal codes linked to the 2016 census data), and rurality (defined by Statistics Canada as living in a rural or small community with a population size <10 000 persons outside the commuting zone of larger urban centers). Among those who received an RAI-HC or RAI-MDS assessment, we also reported the following: marital status, presence of chewing or swallowing problems, relationship with their primary caregiver (only RAI-HC), and do-not-resuscitate (DNR) and do-not-hospitalize (DNH) orders (only RAI-MDS). We also reported their health profiles and functional and cognitive status as measured by the Activities of Daily Living Self-Performance Hierarchy Scale (ADL-H; scores range from 0-6),^33^ the Cognitive Performance Scale (scores range from 0-6),^33^ and the Changes in Health, End-Stage Disease and Signs and Symptoms Scale (CHESS; scores range from 0-5),^33^ which is a measure of health instabilities and an indicator of being at the end of life. Details of these scales have been previously reported and found to be valid^29,30,34,35^ and reliable.^30,34,35^ Higher scores on these measures indicate higher levels of impairment or instability.

### Characteristics of the Hospitalization and Outcomes Post Hospitalization

We examined the following characteristics captured during individuals’ index hospitalization: (1) the proportion admitted to the intensive care unit (ICU) during hospitalization and mean length of stay (in days) in the ICU; (2) among those who received a feeding tube, mean time from admission to receipt of feeding tube and hospital length of stay (in days); and (3) discharge disposition, specifically whether individuals died in hospital or were discharged home, to the ICU, or to inpatient care. We reported 14-, 30-, 90-, 180-, and 360-day mortality rates from admission date of index hospitalization. Among those who survived until discharge from index hospitalization, we examined rates of mortality and rehospitalization or emergency department visits within 1 year post discharge.

### Statistical Analysis

Data were analyzed between October 2021 and November 2024. We used descriptive statistics (ie, mean [SD] and median [IQR] for continuous variables and proportions for categorical variables) to describe the cohort characteristics (ie, individuals’ sociodemographic characteristics, health, and functioning prior to hospitalization) and the main outcomes of our descriptive analyses (hospitalization characteristics and outcomes post hospitalization). We performed sensitivity analyses stratifying these descriptive analyses by home care vs LTC subcohorts and by CHESS scores of 3 or greater (indicative of high health instability) vs less than 3. We performed logistic regression with receipt of feeding tubes as the outcome, separately for subcohorts of older adults who were receiving home care (RAI-HC) or admitted to LTC (RAI-MDS), to examine the associations between patient and care-related characteristics and their likelihood of receiving a feeding tube. Factors included in the regressions were prespecified, that is, chosen if there was evidence of associations with the receipt of feeding tubes in the literature or identified as potentially relevant by clinical experts. For both the home care and LTC subcohorts, we included individuals’ sociodemographic characteristics (eg, sex, age) and their health and functioning (eg, ADL-H, Cognitive Performance Scale) in the modeling. As the RAI-HC and RAI-MDS differed in assessment items, the following factors were included in only one of the models: marital status (only included from the RAI-HC due to the large amount of nonrandom missingness in the RAI-MDS) and DNR and DNH advance directives (only RAI-MDS). Age (in 5-year groups) was entered as a continuous variable since a plot of the restricted cubic spline of age against the logit of the outcome showed a linear association. All factors were kept in the model since no collinearity issues were detected using the SAS VARCLUS procedure.^36^ Statistical tests were 2 tailed with *P* < .05 indicating statistical significance. We also performed sensitivity analyses that added the comorbidities of stroke, cancer, chronic obstructive pulmonary disorder, chronic coronary syndrome, and acute myocardial infarction into both regressions. All analyses were performed using SAS Enterprise, version 9.4 (SAS Institute Inc).^37^

## Results

### Cohort Characteristics

During the study period, 143 331 individuals with dementia met the inclusion criteria (83 536 [58.3%] female and 59 795 [41.7%] male; mean [SD] age, 83.8 [7.5] years). Table 1 describes the characteristics of our cohort, among whom 1312 (0.9%) received a PEG feeding tube insertion during their hospitalization. Feeding tube recipients were younger than nonrecipients (mean [SD] age, 80.8 [7.6] vs 83.8 [7.5] years). Male individuals were more likely than female individuals to receive a feeding tube (722 [1.2%] vs 590 [0.7%]). Residents in urban settings were also more likely to receive a feeding tube than those in rural settings (1238 [1.0%] vs 67 [0.4%]). The proportions of individuals with various comorbidities based on receipt of a feeding tube are provided in eTable 4 in Supplement 1. The main reasons for hospitalization among feeding tube recipients are provided in eTable 5 in Supplement 1.

**Table 1.** Receipt of Feeding Tube Among Hospitalized Patients With Dementia by Sociodemographic Characteristics

Characteristic	Feeding tube receipt, No. (%)^a^		Total No. of patients (n = 143 331)
	Nonrecipient (n = 142 019 [99.1%])	Recipient (n = 1312 [0.9%])	
Age at admission, y			
Mean (SD)	83.8 (7.5)	80.8 (7.6)	83.8 (7.5)
Median (IQR)	85.0 (79.0-89.0)	82.0 (75.0-86.0)	85.0 (79.0-89.0)
Sex			
Female	82 946 (99.3)	590 (0.7)	83 536 (100)
Male	59 073 (98.8)	722 (1.2)	59 795 (100)
Time from dementia diagnosis to admission, y			
Mean (SD)	3.5 (3.6)	3.9 (3.9)	3.5 (3.6)
Median (IQR)	2.3 (0.7-5.2)	2.7 (0.8-5.9)	2.3 (0.7-5.2)
Neighborhood income quintile^b^			
Lowest	37 067 (99.0)	364 (1.0)	37 431 (100)
Low	31 813 (99.1)	295 (0.9)	32 108 (100)
Middle	26 565 (99.0)	255 (1.0)	26 820 (100)
High	23 489 (99.2)	191 (0.8)	23 680 (100)
Highest	22 228 (99.1)	200 (0.9)	22 428 (100)
Rurality^b^			
Rural	15 372 (99.6)	67 (0.4)	15 439 (100)
Urban	125 832 (99.0)	1238 (1.0)	127 070 (100)

^a^ Percentages are based on row total number.

^b^ Owing missing values for this characteristic (<0.1%), totals are less than numbers in column headings.

### Characteristics of Feeding Tube Recipients in Home Care or in LTC

Table 2 shows the sociodemographic, health, and care-related characteristics of individuals residing in the community and receiving home care or LTC at the time of hospitalization who received a feeding tube. Among 42 441 home care recipients (ie, assessed with the RAI-HC), 383 (0.9%) received a feeding tube, of whom 101 (26.4%) had a moderate level of ADL impairment and 109 (28.5%) had a maximal level (indicating moderate to high levels of functional dependency), and 217 (56.7%) had moderate to total cognitive impairment. Nearly one-quarter of these individuals had moderate to high health instability (90 [23.5%]) as indicated by a CHESS score of 3 or greater; about one-third were widowed, separated, or divorced (140 [36.6%]); and most relied on a spouse (178 [46.5%]) or a child (168 [43.9%]) as their primary caregiver and did not have chewing (335 [87.5%]) or swallowing problems (218 [56.9%]) prior to hospitalization.

**Table 2.** Characteristics of Subcohorts of Patients With Dementia Within 6 Months Prior to Hospitalization Who Received a Feeding Tube

Characteristic	Subcohort, No. (%)	
	RAI-HC (n = 383)	RAI-MDS (n = 452)
ADL-H score^a^		
0 (Independent)	60 (15.7)	<6
1-2	113 (29.5)	26-30 (5.8-6.6)^b^
3-4	101 (26.4)	158 (35.0)
5-6 (Need maximal assistance or fully dependent)	109 (28.5)	129 (28.5)
CHESS score^a^		
0 (Most stable)	70 (18.3)	189 (41.8)
1-2	223 (58.2)	233 (51.5)
≥3	90 (23.5)	30 (6.6)
CPS^a^		
0 (Cognitively intact)	16 (4.2)	22 (4.9)
1-2	150 (39.2)	88 (19.5)
≥3	217 (56.7)	342 (75.7)
Marital status		
Missing or unknown	<6	265 (58.6)
Never married	10-15 (2.6-3.9)^b^	12 (2.7)
Separated or divorced	27 (7.0)	22 (4.9)
Widowed	113 (29.5)	66 (14.6)
Married	229 (59.8)	87 (19.2)
Primary caregiver’s relationship to patient		
No caregiver	8 (2.1)	NA
Child or child-in-law	168 (43.9)	NA
Spouse	178 (46.5)	NA
Other relative	15 (3.9)	NA
Friend or neighbor	14 (3.7)	NA
DNR directive		
Missing	NA	16 (3.5)
Present	NA	229 (50.7)
None	NA	207 (45.8)
DNH directive		
Missing	NA	16 (3.5)
Present	NA	76 (16.8)
None	NA	360 (79.6)
Chewing problems		
Present	48 (12.5)	294 (65.0)
None	335 (87.5)	158 (35.0)
Swallowing problems		
Present	165 (43.1)	229 (50.7)
None	218 (56.9)	223 (49.3)

Abbreviations: ADL-H, Activities of Daily Living Self-Performance Hierarchy Scale; CHESS, Changes in Health, End-Stage Disease and Symptoms and Signs scale; CPS, Cognitive Performance Scale; DNH, do not hospitalize; DNR, do not resuscitate; NA, not applicable; RAI-HC, Resident Assessment Instrument in Home Care; RAI-MDS, Resident Assessment Instrument in Long-Term Care.

^a^ Values were missing for less than 0.1%.

^b^ Range reported to prevent presentation of exact numbers less than 6.

Among the 38 028 LTC residents (ie, assessed with the RAI-MDS), 452 (1.2%) received a feeding tube, of whom 158 (35.0%) had moderate levels of ADL impairment and 129 (28.5%) had maximal levels, and 342 (75.7%) had moderate to total cognitive impairment. Only 30 (6.6%) had moderate to high health instability (CHESS score of ≥3). Prior to hospitalization, about one-third of these individuals did not have chewing problems (158 [35.0%]), and half did not have swallowing problems (223 [49.3%]). A total of 229 (50.7%) had in place DNR advanced directives and 76 (16.8%) had in place DNH advanced directives.

### Inpatient Hospitalization Characteristics

Table 3 shows the characteristics of the hospitalization among all individuals in our study, by receipt of feeding tube. Feeding tube recipients (compared with nonrecipients) were 4 times more often admitted into the ICU (557 [42.5%] vs 14 423 [10.2%]), stayed 6 times longer in the ICU (mean [SD], 26.8 [48.0] vs 4.3 [8.1] days), and stayed more than 4 times longer in hospital (mean [SD], 65.6 [120.8] vs 14.8 [35.2] days). Deaths in hospital occurred in 294 feeding tube recipients (22.4%), compared with 14 698 nonrecipients (10.3%). Within 1 year of hospital admission, 743 feeding tube recipients (56.6%) died, compared with 49 987 nonrecipients (35.2%).

**Table 3.** Inpatient Hospitalization Characteristics and Outcomes Among Individuals With Dementia By Feeding Tube Receipt

Characteristic	Feeding tube receipt		
	Nonrecipient (n = 142 019)	Recipient (n = 1312)	All (n = 143 331)
Time from admission to feeding tube insertion, d			
Mean (SD)	NA	25.7 (62.1)	25.7 (62.1)
Median (IQR)	NA	14.0 (6.0-27.0)	14 (6.0-27.0)
Admitted to ICU during hospitalization, No. (%)	14 423 (10.2)	557 (42.5)	14 980 (10.5)
Time from admission to ICU entry, d			
Mean (SD)	2.9 (12.8)	7.6 (33.4)	3.1 (14.1)
Median (IQR)	1.0 (1.0-2.0)	1.0 (1.0-3.0)	1.0 (1.0-3.0)
Length of stay in ICU, d			
Mean (SD)	4.3 (8.1)	26.8 (48.0)	5.1 (12.9)
Median (IQR)	2.7 (1.2-4.9)	11.8 (4.9-29.9)	2.7 (1.2-5.1)
Length of stay in hospital, d			
Mean (SD)	14.8 (35.2)	65.6 (120.8)	15.2 (37.2)
Median (IQR)	7.0 (4.0-14.0)	28.0 (16.0-63.0)	7.0 (4.0-14.0)
Discharge disposition, No. (%)			
Died in hospital	14 698 (10.3)	294 (22.4)	14 992 (10.5)
Discharged home	73 184 (51.5)	337 (25.7)	73 521 (51.3)
Discharged to institutional care	52 762 (37.2)	660 (50.3)	53 422 (37.3)
Discharged to inpatient care	492 (0.3)	14 (1.1)	506 (0.4)
Mortality rates from admission date, No. (%)			
14-d	14 535 (10.2)	62 (4.7)	14 597 (10.2)
30-d	20 630 (14.5)	175 (13.3)	20 805 (14.5)
90-d	30 530 (21.5)	430 (32.8)	30 960 (21.6)
180-d	38 554 (27.1)	579 (44.1)	39 133 (27.3)
360-d	49 987 (35.2)	743 (56.6)	50 730 (35.4)

Abbreviations: ICU, intensive care unit; NA, not applicable.

### Mortality and Rehospitalization Patterns After Discharge

Table 4 and the eFigure in Supplement 1 show the mortality and rehospitalization patterns of survivors of the hospitalization. Mortality and rehospitalization rates were consistently higher among feeding tube recipients at all time points within 1 year of discharge from hospital. At 360 days post discharge, 509 of 1018 feeding tube recipients (50.0%) had died, compared with 36 162 of 127 321 nonrecipients (28.4%), while 512 of 1018 feeding tube recipients (50.3%) were rehospitalized, compared with 54 575 of 127 321 nonrecipients (43.0%). Sensitivity analyses on mortality and rehospitalization for the home care and LTC subcohorts as well as by CHESS scores showed similar patterns (eTables 6-9 in Supplement 1).

**Table 4.** Mortality and Rehospitalization Patterns Post Discharge Among Those Who Survived Hospitalization, by Feeding Tube Receipt

Outcome	Feeding tube receipt, No. (%)		
	Nonrecipient (n = 127 321)	Recipient (n = 1018)	All (n = 128 339)
Mortality rates from discharge date			
30-d	9920 (7.8)	149 (14.6)	10 069 (7.8)
90-d	17 754 (13.9)	265 (26.0)	18 019 (14.0)
180-d	25 099 (19.7)	370 (36.3)	25 469 (19.8)
360-d	36 162 (28.4)	509 (50.0)	36 671 (28.6)
Mortality rates from feeding tube insertion			
30-d	NA	289 (22.0)	NA
90-d	NA	483 (36.8)	NA
180-d	NA	605 (46.1)	NA
360-d	NA	756 (57.6)	NA
Rehospitalization			
30-d	16 470 (12.9)	202 (19.8)	16 672 (13.0)
90-d	29 689 (23.3)	340 (33.4)	30 029 (23.4)
180-d	41 008 (32.2)	416 (40.9)	41 424 (32.3)
360-d	54 757 (43.0)	512 (50.3)	55 269 (43.1)
Unplanned ED admission			
30-d	27 180 (21.3)	280 (27.6)	27 460 (21.4)
90-d	46 830 (36.8)	472 (46.4)	47 302 (36.9)
180-d	62 252 (48.9)	563 (55.3)	62 815 (48.9)
360-d	78 642 (61.8)	659 (64.7)	79 301 (61.8)

Abbreviations: ED, emergency department; NA, not applicable.

### Multivariable Analysis

Table 5 presents results of 2 logistic regression models (model 1 among home care recipients; model 2 among LTC residents) examining factors associated with the likelihood of receiving a feeding tube in hospital. Among home care recipients, being female (odds ratio [OR], 0.66; 95% CI, 0.52-0.84), older (OR for every 5-year increase in age, 0.75; 95% CI, 0.70-0.81), widowed (OR, 0.66; 95% CI, 0.47-0.94), and living in rural settings (OR, 0.38; 95% CI, 0.22-0.66) were associated with reduced odds of receiving a feeding tube. In contrast, having an ADL-H score of 5 or 6 (OR, 2.75; 95% CI, 1.80-4.20) or swallowing problems (OR, 2.22; 95% CI, 1.99-2.49) was associated with higher odds of receiving a feeding tube. Similar patterns were observed for LTC residents. Being female (OR, 0.77; 95% CI, 0.63-0.94), older (OR for every 5-year increase in age, 0.82; 95% CI, 0.77-0.87), and living in rural settings (OR, 0.51; 95% CI, 0.31-0.83) also were associated with reduced odds of receiving a feeding tube, while having an ADL-H score of 5 or 6 (OR, 3.52; 95% CI, 1.10-11.27) or swallowing problems (OR, 2.29; 95% CI, 1.81-2.89) were associated with increased odds. Additionally, having a DNR order was associated with reduced odds of receiving a feeding tube (OR, 0.38; 95% CI, 0.31-0.47), while having chewing problems was associated with increased odds (OR, 1.88; 95% CI, 1.47-2.40). Other factors were not associated with and did not increase the likelihood of receiving a feeding tube. Our sensitivity analyses (eTable 10 in Supplement 1) showed that none of the comorbidities was statistically significant, nor did the comorbidities impact the associations with other factors.

**Table 5.** Multivariable Logistic Regression Models to Examine the Factors Associated With Feeding Tube Receipt

Characteristic	Subgroup, OR (95% CI)	
	RAI-HC^a^	RAI-MDS^b^
Sex		
Male	1 [Reference]	1 [Reference]
Female	0.66 (0.52-0.84)	0.77 (0.63-0.94)
Age in 5-y increments	0.75 (0.70-0.81)	0.82 (0.77-0.87)
Marital status		
Married	1 [Reference]	NA
Divorced/separated	0.71 (0.43-1.19)	NA
Never married	0.90 (0.43-1.88)	NA
Other	0.17 (0.02-1.25)	NA
Widowed	0.66 (0.47-0.94)	NA
Income		
Lowest	1 [Reference]	1 [Reference]
Low	1.25 (0.92-1.68)	0.86 (0.66-1.13)
Middle	1.15 (0.84-1.59)	1.08 (0.83-1.42)
High	0.95 (0.67-1.35)	0.79 (0.58-1.10)
Highest	0.87 (0.61-1.25)	1.00 (0.73-1.37)
Rurality		
Urban	1 [Reference]	1 [Reference]
Rural	0.38 (0.22-0.66)	0.51 (0.31-0.83)
Primary caregiver’s relationship		
Spouse	1 [Reference]	NA
Child or child-in-law	1.28 (0.93-1.79)	NA
Other relative	0.78 (0.41-1.50)	NA
Friend or neighbor	1.32 (0.69-2.56)	NA
ADL-H score		
0	1 [Reference]	1 [Reference]
1-2	1.24 (0.88-1.73)	1.48 (0.45-4.95)
3-4	1.47 (1.02-2.12)	1.90 (0.60-6.05)
5-6	2.75 (1.80-4.20)	3.52 (1.10-11.27)
CHESS score		
0	1 [Reference]	1 [Reference]
1-2	0.95 (0.71-1.27)	0.98 (0.80-1.20)
3+	0.79 (0.57-1.10)	0.79 (0.53-1.19)
CPS score		
0	1 [Reference]	1 [Reference]
1-2	0.59 (0.34-1.10)	1.26 (0.78-2.03)
3-4	0.76 (0.43-1.33)	1.03 (0.65-1.63)
5-6	0.78 (0.44-1.40)	1.31 (0.81-2.11)
Has DNR directive		
No	NA	1 [Reference]
Yes	NA	0.38 (0.31-0.47)
Has DNH directive		
No	NA	1 [Reference]
Yes	NA	1.02 (0.78-1.34)
Has swallowing problems		
No	1 [Reference]	1 [Reference]
Yes	2.22 (1.99-2.49)	2.29 (1.81-2.89)
Has chewing problems		
No	1 [Reference]	1 [Reference]
Yes	0.78 (0.57-1.09)	1.88 (1.47-2.40)

Abbreviations: ADL-H, Activities of Daily Living Self-Performance Hierarchy Scale; CHESS, Changes in Health, End-Stage Disease and Symptoms and Signs scale; CPS, Cognitive Performance Scale; DNH, do not hospitalize; DNR, do not resuscitate; NA, not applicable; OR, odds ratio; RAI-HC, Resident Assessment Instrument in Home Care; RAI-MDS, Resident Assessment Instrument in Long-Term Care.

^a^ Excluded 1072 people (2.5%) from regression analysis as they had missing values for at least one of the covariates in the model.

^b^ Excluded 2362 people (3.2%) from regression analysis as they had missing values for at least one of the covariates in the model.

## Discussion

Our population-based retrospective cohort study examined characteristics associated with receipt of a feeding tube among hospitalized older adults with dementia in Ontario. Overall, 0.9% of hospitalized individuals with dementia received a feeding tube, which is similar to previous findings on the incidence of feeding tube use among individuals with advanced dementia.^38^ Compared with a study on older adults at a geriatric medical center in the US that found the incidence of enteral feeding use to be 1.35%,^38^ the incidence in our study of individuals with dementia was lower, possibly in (at least partial) compliance with guidelines. Feeding tube recipients were 4 times more likely to have been admitted to the ICU, had a mean length of stay of longer than 2 months (4 times longer than nonrecipients), and were more than twice as likely to die in hospital than nonrecipients, suggesting that feeding tube recipients likely had greater frailty than nonrecipients. We found that large proportions of feeding tube recipients had severe cognitive and functional impairments, and more than half of those in LTC also had a DNH order prior to admission. These findings show that a substantial portion of feeding tube recipients were likely incapable of making decisions, leaving decision making to their family caregivers and physicians. Past studies have shown that, despite guidelines and clinical judgments against feeding tube insertion, substitute decision-makers and even physicians continue to hold misperception of the efficacy of tube feeding in improving patient outcomes.^39,40,41,42^ This is exacerbated by the lack of education and conversations on feeding tube use or other options for patients and their families^21,39^; a study on family caregivers of individuals who died with dementia^39^ showed that more than half of the caregivers reported no or less than 15 minutes of discussion regarding feeding tube insertion. Physicians also often experience a lack of control in this decision.^39^ Most physicians surveyed in a study by Gieniusz et al^43^ stated that families often request feeding tube insertion even when physicians counsel against it, and many physicians have concerns about potential litigation. Advanced care planning and education on possible adverse results and alternatives for feeding are particularly important and recommended for these individuals and their families.^44,45^

Consistent with most previous studies,^11,12,14,15,16,17,18,19,21,46^ most of which focused on patients with advanced dementia, our study found no survival benefit of feeding tube insertion in hospitalized patients with dementia, even without restricting to an advanced dementia stage. We found that older adults with dementia who received a feeding tube had higher rates of mortality, rehospitalization, and admission to the emergency department post discharge, in both the short and long term, which concur with past findings.^9,10,13,15,20,23,47^ Given high and increased rates of disability and mortality, questions of appropriateness—including consideration of goal-concordant care—should be explored prior to feeding tube insertion in hospitalized patients with dementia. While we acknowledge that a feeding tube may be the appropriate intervention in some cases, the consistently demonstrated lack of benefits to survival and health outcomes for feeding tube recipients suggests that they may benefit more through a holistic palliative care approach rather than this invasive intervention.^1,13^ Appropriate less invasive alternatives do exist, such as assisted feeding interventions (eg, hand feeding, postural changes to head and/or body position, dietary modification including specialized dysphagia diet or high-calorie supplement),^48^ which constitute a part of palliative care.^49^ Focused education for the clinical care teams, as well as individualized and patient-centered discussions, could help individuals and their families make informed decisions about appropriate care options. There may also be opportunities for protocol changes in clinical settings. For example, referrals for feeding tube insertion for individuals with dementia could automatically trigger a palliative care consultation to initiate the aforementioned discussions and establish goals of care.

We also found that being male, having high functional impairment, being younger, and having swallowing problems were associated with increased odds of receiving a feeding tube, while having a DNR order and living in rural areas were associated with reduced odds. Some findings are consistent with previous studies; for example, it has previously been reported that prehospitalization dysphagia increased the odds of feeding tube placement.^50^ Given that some variables (eg, sex, urbanicity) should not be associated with medical indications for feeding tube insertion, further consideration should be given to the potential impacts of sociocultural considerations and system-level barriers (eg, constraints on access to necessary resources and medical care in rural settings) on clinical decision-making processes. Furthermore, we found no association for some factors that could be expected to impact feeding tube insertion (eg, level of cognitive impairment, DNH order). This points to a potential need for the use of decision aids to enable informed decision making by both clinicians and patients and/or their caregivers. There is clear evidence that decision aids can help make information explicit, provide information about options and their associated benefits and harms, and identify congruence between decisions and personal values.^51^

### Limitations

To our knowledge, this study is the first to use comprehensive population-based health care data to examine factors and outcomes associated with receiving a feeding tube among all hospitalized patients with dementia in Ontario. However, our study has several limitations. First, our regression models, which required data from RAI assessments, were only performed on data from individuals in home care or LTC. While this may have limited the generalizability of our findings to the broader population with dementia, this allowed us to examine many factors associated with individuals’ health, functioning, and care needs that were previously not addressed. We also acknowledge there may be residual confounding in our modeling; for example, we did not have data on individuals’ race or ethnicity, which was previously identified to be associated with receipt of a feeding tube.^12^ A recent study^52^ also demonstrated that racial and cultural differences exist in the perception of feeding tube use, including misconceptions about their effectiveness and the clinical course of dementia. Finally, there may be misclassifications of individuals’ functional, health, and care-related status, since the assessments were conducted within 6 months of hospitalization and these statuses could have changed in the time between assessment and hospitalization. We note this limitation since a sudden change in these statuses, such as developing chewing and swallowing problems or a rapid deterioration in health, could have provoked the hospitalization. Such misclassification would likely bias the effects of these factors toward the null, which could result in factors such as higher CHESS scores to seemingly have no effect on receipt of a feeding tube.

## Conclusions

In this cohort study of hospitalized individuals with dementia in Ontario, only 0.9% received a feeding tube; however, this still represented more than 1000 insertions. Many feeding tube recipients showed high levels of cognitive and functional impairments and had higher mortality and rehospitalization rates than those who did not receive a feeding tube. We found no associations for certain factors that could be expected to have impacted feeding tube insertion, while factors such as sex and urbanicity were associated. These findings showed a clear need for effective and timely conversations around goals of care and alternate intervention options, improved policy and clinical protocols, and potential use of decision aids to help the care team as well as the patients and their families make informed decisions.

## Notes

Supplement 1. **eMethods.** Dementia Case Definition **eTable 1.** Descriptions of Databases **eTable 2.** Description of the RAI-HC and RAI-MDS Variables Used to Assess Individuals’ Functional Status, Health Profiles, Service Use, Advance Directives, and Caregiver Information **eTable 3.** CCI and OHIP Fee Codes Used to Identify Receipt of Feeding Tube During Hospitalization **eTable 4.** Receipt of Feeding Tube Among Hospitalized Patients With Dementia According to Status of Prevalent Comorbidities **eTable 5.** Top 10 MRDX for Those With Feeding Tube Insertion During Index (n = 1312) **eFigure.** Cumulative Incidence of Death During Hospitalization (Left) and Following Hospital Discharge (Right), According to Receipt of Feeding Tube **eTable 6.** Mortality and Rehospitalization Patterns Post Discharge Among Those Receiving Home Care in the Community Who Survived Hospitalization, by Receipt of Feeding Tube **eTable 7.** Mortality and Rehospitalization Patterns Post Discharge Among Those Residing in Long-Term Care Homes Who Survived Hospitalization, by Receipt of Feeding Tube **eTable 8.** Mortality and Rehospitalization Patterns Post Discharge Among Those With CHESS Scores of 3 or Higher Who Survived Hospitalization, by Receipt of Feeding Tube **eTable 9.** Mortality and Rehospitalization Patterns Post Discharge Among Those With CHESS Scores of Less Than 3 Who Survived Hospitalization, by Receipt of Feeding Tube **eTable 10.** Sensitivity Analyses Assessing the Effect of Adding Comorbidities to Multivariable Logistic Regression Models Examining the Factors Associated With Receipt of a Feeding Tube

Supplement 2.
Data Sharing Statement
