# Supplementary material for: Use of Feeding Tubes Among Hospitalized Older Adults With Dementia
Source: JAMA Netw Open. 2025 Feb 20;8(2):e2460780. doi: 10.1001/jamanetworkopen.2024.60780 (PMC11843365; doi:10.1001/jamanetworkopen.2024.60780)
Supplement: Supplement 1. — eMethods. Dementia Case Definition eTable 1. Descriptions of Databases eTable 2. Description of the RAI-HC and RAI-MDS Variables Used to Assess Individuals’ Functional Status, Health Profiles, Service Use, Advance Directives, and Caregiver Information eTable 3. CCI and OHIP Fee Codes Used to Identify Receipt of Feeding Tube During Hospitalization eTable 4. Receipt of Feeding Tube Among Hospitalized Patients With Dementia According to Status of Prevalent Comorbidities eTable 5. Top 10 MRDX for Those With Feeding Tube Insertion During Index (n = 1312) eFigure. Cumulative Incidence of Death During Hospitalization (Left) and Following Hospital Discharge (Right), According to Receipt of Feeding Tube eTable 6. Mortality and Rehospitalization Patterns Post Discharge Among Those Receiving Home Care in the Community Who Survived Hospitalization, by Receipt of Feeding Tube eTable 7. Mortality and Rehospitalization Patterns Post Discharge Among Those Residing in Long-Term Care Homes Who Survived Hospitalization, by Receipt of Feeding Tube eTable 8. Mortality and Rehospitalization Patterns Post Discharge Among Those With CHESS Scores of 3 or Higher Who Survived Hospitalization, by Receipt of Feeding Tube eTable 9. Mortality and Rehospitalization Patterns Post Discharge Among Those With CHESS Scores of Less Than 3 Who Survived Hospitalization, by Receipt of Feeding Tube eTable 10. Sensitivity Analyses Assessing the Effect of Adding Comorbidities to Multivariable Logistic Regression Models Examining the Factors Associated With Receipt of a Feeding Tube [file jamanetwopen-e2460780-s001.pdf]

Supplementary Online Content

Hartford A-M, Li W, Qureshi D, et al. Use of feeding tubes among hospitalized older adults with dementia. *JAMA Netw Open*. 2025;8(2):e2460780. doi:10.1001/jamanetworkopen.2024.60780

eMethods. Dementia Case Definition

eTable 1. Descriptions of Databases

eTable 2. Description of the RAI-HC and RAI-MDS Variables Used to Assess Individuals' Functional Status, Health Profiles, Service Use, Advance Directives, and Caregiver Information

eTable 3. CCI and OHIP Fee Codes Used to Identify Receipt of Feeding Tube During Hospitalization

eTable 4. Receipt of Feeding Tube Among Hospitalized Patients With Dementia According to Status of Prevalent Comorbidities

eTable 5. Top 10 MRDX for Those With Feeding Tube Insertion During Index (n = 1312)

eFigure. Cumulative Incidence of Death During Hospitalization (Left) and Following Hospital Discharge (Right), According to Receipt of Feeding Tube

eTable 6. Mortality and Rehospitalization Patterns Post Discharge Among Those Receiving Home Care in the Community Who Survived Hospitalization, by Receipt of Feeding Tube

eTable 7. Mortality and Rehospitalization Patterns Post Discharge Among Those Residing in Long-Term Care Homes Who Survived Hospitalization, by Receipt of Feeding Tube

eTable 8. Mortality and Rehospitalization Patterns Post Discharge Among Those With CHESS Scores of 3 or Higher Who Survived Hospitalization, by Receipt of Feeding Tube

eTable 9. Mortality and Rehospitalization Patterns Post Discharge Among Those With CHESS Scores of Less Than 3 Who Survived Hospitalization, by Receipt of Feeding Tube

eTable 10. Sensitivity Analyses Assessing the Effect of Adding Comorbidities to Multivariable Logistic Regression Models Examining the Factors Associated With Receipt of a Feeding Tube

This supplementary material has been provided by the authors to give readers additional information about their work.

© 2025 Hartford A-M et al. *JAMA Network Open*.

eMethods. Dementia Case Definition

Case definition- Dementia diagnosis prior to index hospital admission defined as:

- A person aged 40 to 110 years old is identified with dementia if s/he meets one of the following criteria:
    - The person had at least **3** OHIP claims with a dementia diagnosis recorded which were each **at least 30 days apart in a 2-year period**, **or**
    - The person had at least **one** hospitalization or same day surgery with a dementia diagnosis recorded, **or**
    - The person had at least **one** ODB claim with a dementia medication (SUBCLNAM= CHOLINESTERASE INHIBITORS) dispensed

- **OHIP**: 290, 331, 797
- **CIHI ICD 9**: 290.0, 290.1, 290.3, 290.4, 290.8, 290.9, 294.1, 294.8, 294.9, 331.0, 331.1, 331.2, 797.0
- **CIHI ICD 10**: F00.0, F00.1, F00.2, F00.9, F01.0, F01.1, F01.2, F01.3, F01.8, F01.9, F02.0, F02.1, F02.2, F02.3, F02.4, F02.8, F03.X, F05.1, F06.5, F06.6, F06.8, F06.9, F09.X, G300.0, G30.1, G30.8, G30.9, G31.0 G31.1, R54.X

© 2025 Hartford A-M et al. *JAMA Network Open*.

eTable 1. Descriptions of Databases

Database	Description
Registered Persons Database (RPDB)	Contains the demographic information of all Ontario residents with valid health card, including age, sex, date of death, and postal code (used to obtain neighborhood rurality and income quintile).
Discharge Abstract Database (DAD) – Canadian Institute for Health Information (CIHI)	Contains clinical, administrative, and demographic information for each hospital admission.
National Ambulatory Care Reporting System Database (NACRS)	Provides emergency department ambulatory care information.
Same Day Surgery Database (SDS) – Canadian Institute for Health Information (CIHI)	Contains administrative, clinical (diagnoses and procedures), demographic, and administrative information for all patient visits made to day surgery institutions in Ontario.
Ontario Health Insurance Plan Claims Database (OHIP)	Contains health care billing information made by physicians or other health care providers for service reimbursement. This database includes information on the associated diagnosis (i.e., reason for the visit), type of service received, date of the service provided, and the associated billing code.
Ontario Drug Benefit Claims (ODB)	Contains data on all prescription drugs for those eligible for the ODB program (including those over 65 years, on social assistance, residents of LTC, home care recipients, Trillium drug program and special drugs program recipients for those qualifying for assistance). Data includes prescriptions filled and amount prescribed.
Resident Assessment Instrument-Home Care (RAI-HC)	Since 2002, all Ontario residents receiving, or applying to receive, long-term home care or applying for admission into publicly-funded long-term care homes are assessed by the Resident Assessment Instrument-Home Care (RAI-HC). This standardized and routinely administered assessment records information on person’s physical, cognitive, and social functions, service use, and their caregivers’ characteristics and caregiving profile.
Resident Assessment Instrument-Minimum Data Set (RAI-MDS), collected through the Continuing Care Reporting System	Contains the demographic, administrative, and health and functional information for all persons residing in publicly-funded long-term care homes in Ontario. In addition to informing care-planning of residents, the RAI-MDS has been systematically used by Ontario Ministry of Health and Long-Term Care to assess the overall case-mix of facilities and to determine the resource utilization intensity of its residents[3].

© 2025 Hartford A-M et al. *JAMA Network Open*.

eTable 2. Description of the RAI-HC and RAI-MDS Variables Used to Assess Individuals' Functional Status, Health Profiles, Service Use, Advance Directives, and Caregiver Information

Characteristics	RAI-HC variable name and categories used	RAI-MDS variable name and categories used
**Activities of Daily Living Self-Performance Scale (ADL-H)**	ADL_SELF<br><br>Values:<br>• 0 = Independent<br>• 1 = Supervision required<br>• 2 = Limited impairment<br>• 3 = Extensive assistance required (I)<br>• 4 = Extensive assistance required (II)<br>• 5 = Dependent<br>• 6 = Total dependence<br><br>Categorized as:<br>• 0 (Independent)<br>• 1-2<br>• 3-4<br>• 5-6 (Need maximal assistance or fully dependent)	ADL_HIERARCHY<br><br>Values:<br>• 0 = Independent<br>• 1 = Supervision required<br>• 2 = Limited impairment<br>• 3 = Extensive assistance required (I)<br>• 4 = Extensive assistance required (II)<br>• 5 = Dependent<br>• 6 = Total dependence<br><br>Categorized as:<br>• 0 (Independent)<br>• 1-2<br>• 3-4<br>• 5-6 (Need maximal assistance or fully dependent)
**Changes in Health, End-Stage Disease and Symptoms and Signs Scale Score (CHESS)**	CHESS_SCALE<br><br>Values:<br>• 0 = no health instability<br>• 1 = minimal health instability<br>• 2 = low health instability<br>• 3 = moderate heath instability<br>• 4/5 = high or very high health stability<br><br>Categorized as:<br>• 0 (Most stable)<br>• 1-2<br>• 3+	CHESS<br><br>Values:<br>• 0 = no health instability<br>• 1 = minimal health instability<br>• 2 = low health instability<br>• 3 = moderate heath instability<br>• 4/5 = high or very high health stability<br><br>Categorized as:<br>• 0 (Most stable)<br>• 1-2<br>• 3+
**Cognitive Performance Scale Score (CPS)**	COGNITIVE_PERFORMANCE_SCALE<br><br>Values:<br>• 0 = Intact<br>• 1 = Borderline intact<br>• 2 = Mild impairment<br>• 3 = Moderate impairment	CPS<br><br>Values:<br>• 0 = Intact<br>• 1 = Borderline intact<br>• 2 = Mild impairment<br>• 3 = Moderate impairment

© 2025 Hartford A-M et al. *JAMA Network Open*.

	• 4 = Moderate/severe impairment<br>• 5 = Severe impairment<br>• 6 = Very severe impairment<br><br>Categorized as:<br>• 0 (Cognitively intact)<br>• 1-2<br>• 3+	• 4 = Moderate/severe impairment<br>• 5 = Severe impairment<br>• 6 = Very severe impairment<br><br>Categorized as:<br>• 0 (Cognitively intact)<br>• 1-2<br>• 3+
**Marital Status**	BB4<br><br>Values:<br>• Never Married<br>• Married<br>• Widowed<br>• Separated<br>• Divorced<br>• Other<br><br>Categorized as:<br>• Divorced/separated<br>• Never married<br>• Other<br>• Widowed<br>• Married	A5<br><br>Values:<br>• Never Married<br>• Married<br>• Widowed<br>• Separated<br>• Divorced<br>• Other<br><br>Categorized as:<br>• Divorced/separated<br>• Never married<br>• Other<br>• Widowed<br>• Married
**Primary Caregiver's Relationship to Patient**	G1FA_RAIHC<br><br>Values:<br>• No caregiver<br>• Child or child-in-law<br>• Spouse<br>• Other Relative<br>• Friend/Neighbour	N/A
**Advanced Directives**		
Do Not Resuscitate (DNR)	N/A	A10B<br><br>Values:<br>• Missing<br>• Yes<br>• No
Do Not Hospitalize (DNH)	N/A	A10C<br><br>Values:<br>• Missing<br>• Yes<br>• No

© 2025 Hartford A-M et al. *JAMA Network Open*.

**Eating Problems**		
Chewing Problems	M1A<br><br>Values:<br>• Yes<br>• No	K1A<br><br>Values:<br>• Yes<br>• No
Swallowing Problems	L3_RAIHC<br><br>Values:<br>• 0 = Normal<br>• 1 = Requires diet modification to swallow solid foods<br>• 2 = Requires modification to swallow solid foods and liquids<br>• 3 = Combined oral and tube feeding<br>• 4 = No oral intake (NPO)<br><br>Categorized as:<br>• Yes = 0<br>• No = 1, 2, 3, or 4	K1B<br><br>Values:<br>• Yes<br>• No

© 2025 Hartford A-M et al. *JAMA Network Open*.

eTable 3. CCI and OHIP Fee Codes Used to Identify Receipt of Feeding Tube During Hospitalization

**Type of Tube**	**Code**
Gastrostomy Tube	1NF53BABC
	1NF53BATS
	1NF53BTQB
	1NF53BTTS
	1NF53DAQB
	1NF53DATS
	1NF53HATS
	1NF53LAQB
	1NF53LATS
	522
	5510
Gastrostomy-Jejunostomy Tube	1NK77EM
	1NK77RQ
	1NK53DATS
	1NK53LATS
Physician Billing Codes (OHIP)	J055 (Percutaneous gastrostomy)
	S118 (Gastrostomy)
	Z532 (Percutaneous endoscopic gastrostomy)
	S134 (Gastroduodenostomy or gastrojejunostomy)
	J063 (Percutaneous jejunostomy)
	Z540 (Intubation of small intestine with or without fluoroscopy)

© 2025 Hartford A-M et al. *JAMA Network Open*.

eTable 4. Receipt of Feeding Tube Among Hospitalized Patients With Dementia According to Status of Prevalent Comorbidities

Characteristic	Feeding Tube not Received	Feeding Tube Received	Total
	142,019 (99.1%)	1,312 (0.9%)	143,331
**Number of Active Comorbidities**			
Mean ± SD	2.7 ± 1.7	2.7 ± 1.8	2.74 ± 1.73
Median (IQR)	2.0 (1.0 - 4.0)	3.0 (1.0-4.0)	2.00 (1.00-4.00)
0-1	36,663 (25.8%)	342 (26.1%)	37,005 (25.8%)
2	34,527 (24.3%)	310 (23.6%)	34,837 (24.3%)
3	29,479 (20.8%)	265 (20.2%)	29,744 (20.8%)
4	19,618 (13.8%)	184 (14.0%)	19,802 (13.8%)
5	11,616 (8.2%)	119 (9.1%)	11,735 (8.2%)
6+	10,116 (7.1%)	92 (7.0%)	10,208 (7.1%)
**Comorbidities**			
Osteoarthritis	26,339 (18.5%)	201 (15.3%)	26,540 (18.5%)
Cancer	17,847 (12.6%)	153 (11.7%)	18,000 (12.6%)
Cardiac Arrythmia	10,424 (7.3%)	89 (6.8%)	10,513 (7.3%)
Mood Disorder	19,589 (13.8%)	196 (14.9%)	19,785 (13.8%)
Other Mental Health Disorder	11,746 (8.3%)	129 (9.8%)	11,875 (8.3%)
Osteoporosis	4,790 (3.4%)	42 (3.2%)	4,832 (3.4%)
Renal Failure	7,974 (5.6%)	74 (5.6%)	8,048 (5.6%)
Stroke	7,364 (5.2%)	91 (6.9%)	7,455 (5.2%)
Chronic Coronary Syndrome	14,280 (10.1%)	117 (8.9%)	14,397 (10.0%)
Acute Myocardial Infarction	11,339 (8.0%)	87 (6.6%)	11,426 (8.0%)
Asthma	4,284 (3.0%)	51 (3.9%)	4,335 (3.0%)
Congestive Heart Failure	9,028 (6.4%)	84 (6.4%)	9,112 (6.4%)
Chronic Obstructive Pulmonary Disorder	5,862 (4.1%)	48 (3.7%)	5,910 (4.1%)
Diabetes	5,405 (3.8%)	52 (4.0%)	5,457 (3.8%)
Hypertension	36,128 (25.4%)	303 (23.1%)	36,431 (25.4%)
Rheumatoid Arthritis	890 (0.6%)	10 (0.8%)	900 (0.6%)

© 2025 Hartford A-M et al. *JAMA Network Open*.

eTable 5. Top 10 MRDX for Those With Feeding Tube Insertion During Index (n = 1312)

Main reason for hospital admission (MRDx)	Percent (n) of Feeding Tube Users
**Pneumonitis due to solids and liquids**	14.1 (185)
**Cerebral infarction**	12.6 (166)
**Other septicaemia**	5.8 (76)
**Respiratory failure, not elsewhere classified**	4.5 (59)
**Dysphagia**	3.0 (40)
**Complications of other internal prosthetic devices, implants and grafts**	2.7 (36)
**Intracerebral haemorrhage**	2.7 (36)
**Pneumonia, organism unspecified**	2.6 (34)
**Delirium, not induced by alcohol and other psychoactive substances**	1.9 (25)
**Intracranial injury**	1.8 (24)

eFigure. Cumulative Incidence of Death During Hospitalization (Left) and Following Hospital Discharge (Right), According to Receipt of Feeding Tube

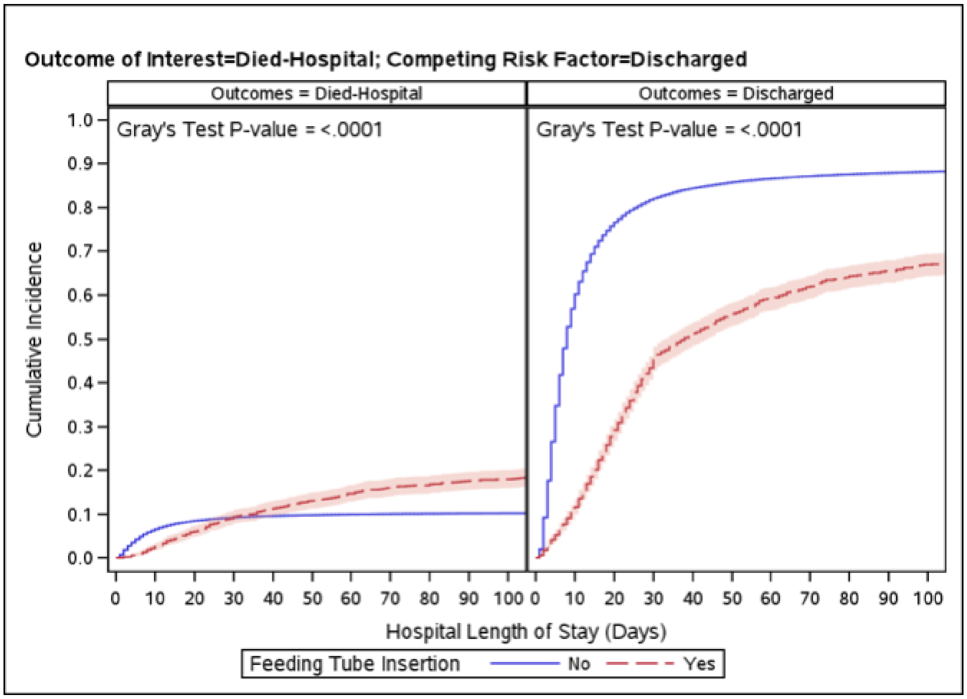

© 2025 Hartford A-M et al. *JAMA Network Open*.

eTable 6. Mortality and Rehospitalization Patterns Post Discharge Among Those Receiving Home Care in the Community Who Survived Hospitalization, by Receipt of Feeding Tube

		**Feeding Tube Not Received** (n=37,402)	**Feeding Tube Received** (n=279)	**Total** (n=37,681)
**Mortality rates from discharge date**	30-day mortality	2,353 (6.3%)	35 (12.5%)	2,388 (6.3%)
	90-day mortality	4,661 (12.5%)	64 (22.9%)	4,725 (12.5%)
	180-day mortality	6,968 (18.6%)	100 (35.8%)	7,068 (18.8%)
	360-day mortality	10,684 (28.6%)	136 (48.7%)	10,820 (28.7%)
**Re-hospitalization**	30-day rehospitalization	5,401 (14.4%)	57 (20.4%)	5,458 (14.5%)
	90-day rehospitalization	9,938 (26.6%)	101 (36.2%)	10,039 (26.6%)
	180-day rehospitalization	13,698 (36.6%)	124 (44.4%)	13,822 (36.7%)
	360-day rehospitalization	18,039 (48.2%)	146 (52.3%)	18,185 (48.3%)
**Unplanned Emergency Department (ED) admission**	30-day ED admission	8,720 (23.3%)	79 (28.3%)	8,799 (23.4%)
	90-day ED admission	15,397 (41.2%)	133 (47.7%)	15,530 (41.2%)
	180-day ED admission	20,218 (54.1%)	159 (57.0%)	20,377 (54.1%)
	360-day ED admission	25,033 (66.9%)	177 (63.4%)	25,210 (66.9%)

© 2025 Hartford A-M et al. *JAMA Network Open*.

eTable 7. Mortality and Rehospitalization Patterns Post Discharge Among Those Residing in Long-Term Care Homes Who Survived Hospitalization, by Receipt of Feeding Tube

		**Feeding Tube Not Received** (n=32,168)	**Feeding Tube Received** (n=391)	**Total** (n=32,559)
**Mortality rates from discharge date**	30-day mortality	5,312 (16.5%)	80 (20.5%)	5,392 (16.6%)
	90-day mortality	8,388 (26.1%)	128 (32.7%)	8,516 (26.2%)
	180-day mortality	10,927 (34.0%)	166 (42.5%)	11,093 (34.1%)
	360-day mortality	14,331 (44.6%)	227 (58.1%)	14,558 (44.7%)
**Re-hospitalization**	30-day rehospitalization	3,729 (11.6%)	79 (20.2%)	3,808 (11.7%)
	90-day rehospitalization	6,335 (19.7%)	128 (32.7%)	6,463 (19.9%)
	180-day rehospitalization	8,564 (26.6%)	157 (40.2%)	8,721 (26.8%)
	360-day rehospitalization	11,106 (34.5%)	193 (49.4%)	11,299 (34.7%)
**Unplanned Emergency Department (ED) admission**	30-day ED admission	5,994 (18.6%)	108 (27.6%)	6,102 (18.7%)
	90-day ED admission	9,709 (30.2%)	179 (45.8%)	9,888 (30.4%)
	180-day ED admission	12,740 (39.6%)	214 (54.7%)	12,954 (39.8%)
	360-day ED admission	15,978 (49.7%)	249 (63.7%)	16,227 (49.8%)

© 2025 Hartford A-M et al. *JAMA Network Open*.

eTable 8. Mortality and Rehospitalization Patterns Post Discharge Among Those With CHESS Scores of 3 or Higher Who Survived Hospitalization, by Receipt of Feeding Tube

		**Feeding Tube Not Received** (n=12,295)	**Feeding Tube Received** (n=85)	**Total** (n=12,380)
**Mortality rates from discharge date**	30-day mortality	1,368 (11.1%)	15 (17.6%)	1,383 (11.2%)
	90-day mortality	2,428 (19.7%)	26 (30.6%)	2,454 (19.8%)
	180-day mortality	3,348 (27.2%)	38 (44.7%)	3,386 (27.4%)
	360-day mortality	4,656 (37.9%)	55 (64.7%)	4,711 (38.1%)
**Re-hospitalization**	30-day rehospitalization	1,774 (14.4%)	18 (21.2%)	1,792 (14.5%)
	90-day rehospitalization	3,246 (26.4%)	31 (36.5%)	3,277 (26.5%)
	180-day rehospitalization	4,404 (35.8%)	36 (42.4%)	4,440 (35.9%)
	360-day rehospitalization	5,668 (46.1%)	46 (54.1%)	5,714 (46.2%)
**Unplanned Emergency Department (ED) admission**	30-day ED admission	2,843 (23.1%)	20 (23.5%)	2,863 (23.1%)
	90-day ED admission	4,972 (40.4%)	38 (44.7%)	5,010 (40.5%)
	180-day ED admission	6,350 (51.6%)	46 (54.1%)	6,396 (51.7%)
	360-day ED admission	7,746 (63.0%)	52 (61.2%)	7,798 (63.0%)

© 2025 Hartford A-M et al. *JAMA Network Open*.

eTable 9. Mortality and Rehospitalization Patterns Post Discharge Among Those With CHESS Scores of Less Than 3 Who Survived Hospitalization, by Receipt of Feeding Tube

		**Feeding Tube Not Received** (n=57,275)	**Feeding Tube Received** (n=585)	**Total** (n=57,860)
**Mortality rates from discharge date**	30-day mortality	6,297 (11.0%)	100 (17.1%)	6,397 (11.1%)
	90-day mortality	10,621 (18.5%)	166 (28.4%)	10,787 (18.6%)
	180-day mortality	14,547 (25.4%)	228 (39.0%)	14,775 (25.5%)
	360-day mortality	20,359 (35.5%)	308 (52.6%)	20,667 (35.7%)
**Re-hospitalization**	30-day rehospitalization	7,356 (12.8%)	118 (20.2%)	7,474 (12.9%)
	90-day rehospitalization	13,027 (22.7%)	198 (33.8%)	13,225 (22.9%)
	180-day rehospitalization	17,858 (31.2%)	245 (41.9%)	18,103 (31.3%)
	360-day rehospitalization	23,477 (41.0%)	293 (50.1%)	23,770 (41.1%)
**Unplanned Emergency Department (ED) admission**	30-day ED admission	11,871 (20.7%)	167 (28.5%)	12,038 (20.8%)
	90-day ED admission	20,134 (35.2%)	274 (46.8%)	20,408 (35.3%)
	180-day ED admission	26,608 (46.5%)	327 (55.9%)	26,935 (46.6%)
	360-day ED admission	33,265 (58.1%)	374 (63.9%)	33,639 (58.1%)

© 2025 Hartford A-M et al. *JAMA Network Open*.

eTable 10. Sensitivity Analyses Assessing the Effect of Adding Comorbidities to Multivariable Logistic Regression Models Examining the Factors Associated With Receipt of a Feeding Tube

		**Odds Ratio (95% Confidence Interval)**	
Characteristic		***Home care Recipients (RAI-HC)****	***Long-term Care Residents (RAI-MDS)*****
**Sex** (ref = male)	Female	0.66 (0.52 - 0.83)	0.82 (0.77 - 0.87)
**Age in 5 years**		0.75 (0.69 - 0.80)	0.77 (0.63 - 0.94)
**Marital Status** (ref = married)	Divorced/separated	0.71 (0.42 - 1.18)	n/a
	Never Married	0.90 (0.43 - 1.88)	n/a
	Other	0.17 (0.02 - 1.23)	n/a
	Widowed	0.66 (0.47 - 0.93)	n/a
**Income** (ref = lowest)	Low	1.25 (0.93 - 1.68)	0.86 (0.66 - 1.13)
	Middle	1.15 (0.84 - 1.59)	1.08 (0.83 - 1.42)
	High	0.96 (0.67 - 1.35)	0.79 (0.57 - 1.10)
	Highest	0.88 (0.61 - 1.25)	1.00 (0.73 - 1.36)
**Rurality** (ref = urban)	Rural	0.38 (0.22 - 0.66)	0.51 (0.31 - 0.83)
**Primary Caregiver's Relationship** (ref = spouse)	Child or child-in-law	1.29 (0.94 - 1.78)	n/a
	Other relative	0.79 (0.41 - 1.51)	n/a
	Friend or neighbour	1.34 (0.69 - 2.59)	n/a
**ADL Self Performance Score** (ref = 0)	1-2	1.24 (0.89 - 1.73)	1.50 (0.45 - 4.99)
	3-4	1.47 (1.02 - 2.12)	1.90 (0.60 - 6.04)
	5-6	2.76 (1.81 - 4.22)	3.52 (1.10 - 11.26)
**CHESS Score** (ref = 0)	1-2	0.95 (0.71 - 1.27)	0.98 (0.80 - 1.20)
	3+	0.79 (0.57 - 1.10)	0.79 (0.53 - 1.19)
**CPS Score** (ref = 0)	1-2	0.59 (0.34 - 1.01)	1.25 (0.78 - 2.02)
	3-4	0.75 (0.43 - 1.32)	1.02 (0.64 - 1.62)
	5-6	0.78 (0.43 - 1.39)	1.30 (0.80 - 2.10)
**Has Do Not Resuscitate Directive** (Ref = No)	Yes	n/a	0.38 (0.31 - 0.47)
**Has Do Not Hospitalize Directive** (Ref = No)	Yes	n/a	1.02 (0.78 - 1.35)
**Has Swallowing problems** (Ref = No)	Yes	2.23 (2.00 - 2.50)	2.28 (1.81 - 2.88)
**Has Chewing Problems** (Ref = No)	Yes	0.79 (0.57 - 1.09)	1.88 (1.47 - 2.40)
**Stroke** (Ref = No)	Yes	0.93 (0.59 - 1.44)	1.21 (0.87 - 1.68)
**Cancer** (Ref = No)	Yes	1.19 (0.88 - 1.62)	1.06 (0.79 - 1.44)
**Chronic Obstructive Pulmonary Disorder** (Ref = No)	Yes	1.08 (0.63 - 1.85)	0.91 (0.58 - 1.43)
**Chronic Coronary Syndrome** (Ref = No)	Yes	1.18 (0.84 - 1.67)	0.81 (0.56 - 1.17)

© 2025 Hartford A-M et al. *JAMA Network Open*.

**Acute Myocardial Infarction** (Ref = No)	Yes	0.81 (0.53 - 1.23)	1.06 (0.72 - 1.56)

Model 1: among homecare (RAI-HC) recipients, Model 2: among long-term care (RAI-MDS) recipients.

© 2025 Hartford A-M et al. *JAMA Network Open*.
